# Microglia and Astrocytes in Alzheimer’s Disease: Significance and Summary of Recent Advances

**DOI:** 10.14336/AD.2023.0907

**Published:** 2024-08-01

**Authors:** Qianting Deng, Chongyun Wu, Emily Parker, Timon Cheng-Yi Liu, Rui Duan, Luodan Yang

**Affiliations:** ^1^Laboratory of Exercise and Neurobiology, School of Physical Education and Sports Science, South China Normal University, Guangzhou 510006, China.; ^2^Laboratory of Regenerative Medicine in Sports Science, School of Physical Education and Sports Science, South China Normal University, Guangzhou 510006, China.; ^3^Laboratory of Laser Sports Medicine, School of Physical Education and Sports Science, South China Normal University, Guangzhou 510006, China.; ^4^Medical College of Georgia at Augusta University, Augusta, GA 30912, USA.

**Keywords:** Alzheimer’s disease, microglia, astrocytes, synaptic pruning, neuroinflammation

## Abstract

Alzheimer’s disease, one of the most common forms of dementia, is characterized by a slow progression of cognitive impairment and neuronal loss. Currently, approved treatments for AD are hindered by various side effects and limited efficacy. Despite considerable research, practical treatments for AD have not been developed. Increasing evidence shows that glial cells, especially microglia and astrocytes, are essential in the initiation and progression of AD. During AD progression, activated resident microglia increases the ability of resting astrocytes to transform into reactive astrocytes, promoting neurodegeneration. Extensive clinical and molecular studies show the involvement of microglia and astrocyte-mediated neuroinflammation in AD pathology, indicating that microglia and astrocytes may be potential therapeutic targets for AD. This review will summarize the significant and recent advances of microglia and astrocytes in the pathogenesis of AD in three parts. First, we will review the typical pathological changes of AD and discuss microglia and astrocytes in terms of function and phenotypic changes. Second, we will describe microglia and astrocytes’ physiological and pathological role in AD. These roles include the inflammatory response, “eat me” and “don’t eat me” signals, Aβ seeding, propagation, clearance, synapse loss, synaptic pruning, remyelination, and demyelination. Last, we will review the pharmacological and non-pharmacological therapies targeting microglia and astrocytes in AD. We conclude that microglia and astrocytes are essential in the initiation and development of AD. Therefore, understanding the new role of microglia and astrocytes in AD progression is critical for future AD studies and clinical trials. Moreover, pharmacological, and non-pharmacological therapies targeting microglia and astrocytes, with specific studies investigating microglia and astrocyte-mediated neuronal damage and repair, may be a promising research direction for future studies regarding AD treatment and prevention.

## Introduction

Alzheimer’s disease (AD), an age-related neurodegenerative disease, is one of the most common forms of dementia [1, 2]. In the early 20th century, Dr. Alois Alzheimer described the first case of AD in a middle-aged lady (Auguste Deter) who progressively lost cognitive function and developed abnormal behavior [3]. The β-amyloid (Aβ) hypothesis is one of the most common hypotheses explaining the initiation and progression of AD. The progressively accumulated extracellular Aβ forms amyloid plaques, inducing neuronal damage, apoptosis, and degeneration [4-6]. In another widely studied hypothesis, the excessive accumulation of hyperphosphorylated tau causes neuronal dysfunction and induces impaired cognitive function [7, 8]. Current therapeutic agents applied in the clinical treatment of AD primarily focus on eliminating the production of Aβ and abnormal tau [3, 9]. Unfortunately, the approved pharmacological therapies for AD only slow disease progression or temporarily improve cognitive function [3, 10].

Recent studies support the essential role of glial cell-involved neuroinflammation in AD pathologies [10-15]. The excessive activation of glial cells, including astrocytes and microglia, induces Aβ accumulation, neuronal damage, synapse loss, and neurodegeneration [16-20]. Glial cells are essential in preserving homeostasis of the central nervous system (CNS) through the generation of myelin sheaths around the axons, the formation of synapses, and the maintenance of physiological levels of neurotransmitters [21, 22]. However, with the progression of AD, the abnormal generation of pro-inflammatory cytokines and chemokines by microglia and astrocytes causes detrimental effects through the disruption of the neuron system, the dysfunction of synapses, and, ultimately, the decline of cognition [23, 24]. Despite numerous compelling research studies investigating the pathological mechanism of AD, effective curative treatments or drugs are still unavailable [9]. Notably, increasing studies suggest that glial cells exert both neuroprotective and neurotoxic roles in the multifaceted neurodegenerative process of AD [21, 23]. Typically, glial cells promote neuronal survival by releasing neuroprotective cytokines, maintaining an adequate supply of glutamate in synapses, and eliminating extra synaptic terminals [25]. In recent years, the role of glial cells in neurodegenerative diseases, especially in AD, has received increasing attention. In this review, we summarize the significance of glial cells in AD pathologies and discuss the new advances in glial cells in AD.

**Figure 1.**
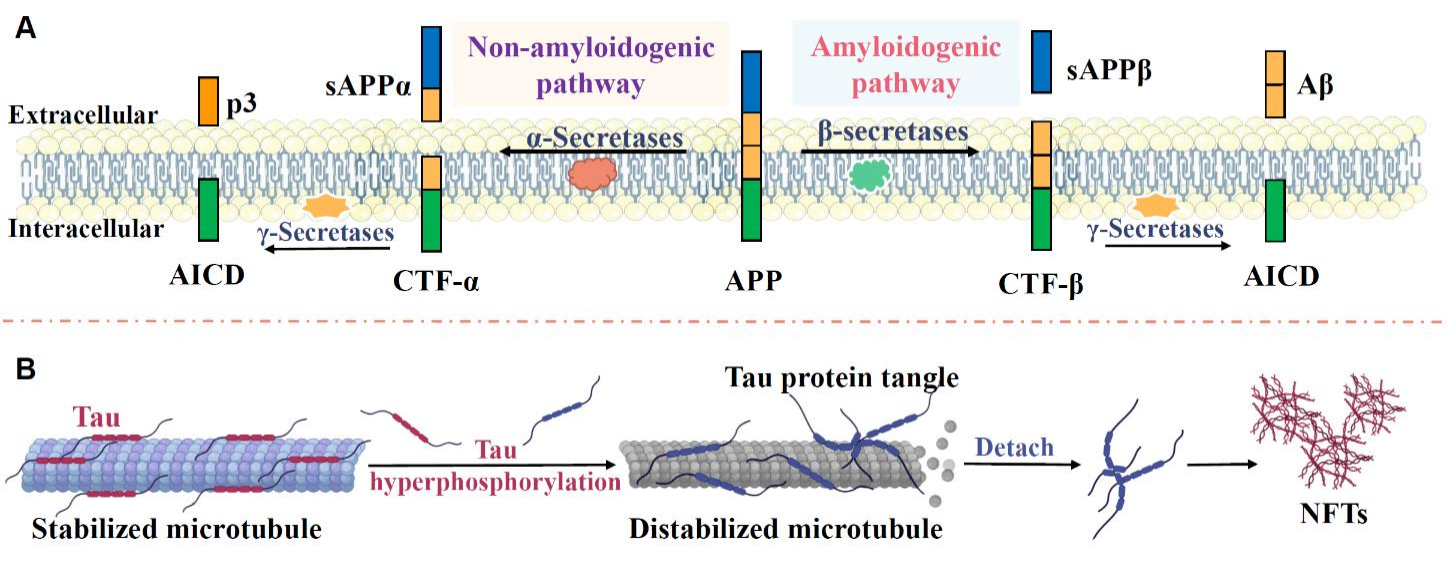
**Typical pathological changes in AD**. (**A**) APP is cleaved by α-, β-, and γ-secretases. Under physiological conditions, APP is mainly cleaved by α-and γ-secretase, which generates a neurotrophic fragment, APPα, and a C-terminal fragment, C83. The C83 is cleaved into the nontoxic peptide P3 and APP intracellular domain (AICD) by γ-secretase. sAPPα and AICD support neurite outgrowth and the formation of learning and memory-related genes, providing neuroprotection for nerve cells. Under pathological conditions, APP is cleaved by β-secretase generating membrane-bound CTFβ fragment and N-terminal APPsβ. CTFβ fragment is then cleaved by γ secretase generating Aβ peptide and the AICD. (**B**) Tau, a microtubule-associated protein, acts as a stabilizer, promoting microtubule stabilization. Under physiological conditions, tau binds to microtubules and maintains cytoskeleton stability, microtubule structure, and neuronal transport. However, under pathological conditions, the abnormally phosphorylated tau protein dissociates from the neuronal microtubules. The detached phosphorylated tau forms paired helical filaments and develops into NFTs.

## Typical pathological changes in AD

*Amyloid plaques*. The amyloid cascade hypothesis was proposed over 30 years ago, posing that AD initiates with Aβ accumulation followed by a series of pathological events resulting in neuronal loss [5, 26]. Aβ is a neurotoxic peptide derived from amyloid precursor protein (APP). As shown in Figure 1A, APP is cleaved by α-, β-, and γ-secretases [27]. Under physiological conditions, APP is mainly cleaved by α-and γ-secretase, which generates a neurotrophic fragment, APPα, and a C-terminal fragment, C83. γ-secretase cleaves C83 into the nontoxic peptide P3 and the APP intracellular domain (AICD) [28, 29]. sAPPα and AICD support neurite outgrowth and the formation of learning and memory-related genes, providing neuroprotection for nerve cells [29, 30]. Under pathological conditions, β-secretase cleaves APP, generating a membrane-bound CTFβ fragment and N-terminal APPsβ. γ secretase cleaves the CTFβ fragment, producing the Aβ peptide and the AICD [29, 31]. The generated Aβ peptide mainly includes Aβ1-40 and the more neurotoxic Aβ1-42 [29, 31]. The extracellular Aβ accumulation induces the formation of neurotoxic amyloid fibrils and the development of insoluble amyloid plaques. These insoluble amyloid plaques cause mitochondrial damage, synaptic dysfunction, oxidative stress, neuroinflammation, neuronal loss, and learning and memory impairment [32, 33]. In addition, the accumulation of Aβ activates Tau protein kinase 1 to enhance the spread of pathological tau, accelerating the development of tau pathology in AD [34].

*Tau pathology.* As shown in Figure 1B, Tau, a microtubule-associated protein, acts as a stabilizer promoting microtubule stabilization. Under physiological conditions, tau binds to microtubules and maintains cytoskeleton stability, microtubule structure, and neuronal transport [35]. The tau hypothesis poses that the hyperphosphorylated tau-induced intracellular neurofibrillary tangles (NFTs) are the pathogenic initiators triggering downstream pathological changes [4]. Under pathological conditions, the abnormally phosphorylated tau protein dissociates from the neuronal microtubules [36, 37]. The detached phosphorylated tau forms paired helical filaments and finally develops into NFTs [36, 37]. The intracellular NFTs cause axonal instability and impair the transport of nutrients and cell signal communication [4].

*Microglia and astrocyte-mediated neuroinflammation in AD.* In addition to Aβ and tau pathologies, neuroinflammation has a prominent role in AD pathology. The activation of microglia, the excessive release of pro-inflammatory cytokines, and the polarization of glial cells contribute to neuroinflammatory changes and exacerbate pathologies [5, 38]. Microglial cells, which arise from marrow myeloid progenitors in the embryonic yolk sac during early development, are the essential innate immune cells accounting for approximately 0.5-16% of the glial cells in the human brain [39, 40]. Based on their morphological and physiological properties, three divisions of microglia exist. These divisions include resting, activated, and phagocytic microglia [41]. Under physiological conditions, microglia are mainly ramified microglia in a resting state. Microglia become activated into an amoeboid state in response to brain injury, misfolded protein, and cell debris in neurodegenerative diseases. During activation, the microglia undergo morphological changes, including the enlargement of cell bodies, the reduction in number and the presence of shorter processes, and the presence of cytoplasmic vacuoles. Functional changes also accompany the morphological changes of microglia [42, 43]. For instance, activated microglia show increases in migration, pro-inflammatory release, and phagocytosis [44]. The phagocytic microglia are full-blown brain macrophages with typical amoeboid morphology and increased release of immune molecules [41].

In AD, microglia express specific pattern recognition receptors (PRRs), including highly conserved pathogen-associated molecular patterns (PAMPs), damage-associated molecular patterns (DAMPs), and nucleotide-binding oligomerization domain (NOD)-like receptors (NLRs), which detect signals near Aβ and stimulate PRRs. The stimulation of PRRs causes the sustained release of neuroinflammatory factors, promoting cell death and exacerbating AD pathologies [43]. Recent studies suggest that TLR4 microglial signaling is critical for mediating the increased production of inflammatory cytokines, the inhibition of phagocytic function, and the accumulation of plaques [42]. Similarly, Aβ peptides activate NLRs, particularly NLR3, promoting the formation of inflammasome complexes and the maturation of IL-1β, leading to subsequent inflammatory events [45]. Moreover, PRRs activate various transcription factors, regulating the expression of several downstream inflammatory response genes [46]. Microglia are also essential in CNS development. During the early stages of brain development, microglia are indispensable in eliminating inappropriate neuronal connections and excess synapses [47]. This process is referred to as synaptic pruning [47]. Excessive synaptic pruning has been detected in AD and is implicated in neuronal damage, loss, and neurodegeneration-associated cognitive deficits [47, 48].

Like microglia, astrocytes are another subtype of glial cells in the CNS that play an essential role in the modulation of neuroinflammation [49]. Under healthy conditions, astrocytes exert various pivotal physiological functions, including synaptogenesis, neurotransmission, ion homeostasis, secretion of growth factor, and blood-brain barrier (BBB) permeability [49-51]. Under pathological conditions, reactive astrocytes undergo morphological and functional changes [42]. These morphological and function changes include hypertrophy of the cell and the overproduction of neurotoxic factors [42]. Characterization of these reactive astrocytes includes increased glial fibrillary acidic protein (GFAP) and vimentin [18]. Increases in reactive astrocytes have been detected around amyloid plaques in AD, suggesting an intimate relationship between astrocytes and typical AD pathologies [18, 52]. Like microglia, astrocytes also detect Aβ aggregates, leading to the activation of target genes and the production of corresponding factors [53]. Furthermore, signal transduction pathways involved in astrocyte responses in AD, including the Janus kinase (JAK)/STAT3, the calcium/calcineurin/NFAT Pathway, the NFκB, and the MAPK pathways, regulate astrocyte APP processing homeostasis and promote overproduction of neurotoxic factors leading to Aβ accumulation and toxicity [54]. However, studies also found that the knockdown GFAP or the attenuation of astrocyte activation accelerates amyloid plaque-associated pathogenesis [55]. This inconsistency may be attributed to the different pathogeneses or pathological stages of AD.

In addition to the three pathogenic mechanisms, several other factors, including cholinergic neuronal dysfunction, vascular impairments, and genetic factors, are implicated in AD’s pathogenesis [56]. Acetylcholine is an essential neurotransmitter that regulates critical physiological processes such as attention, learning, memory, stress response, wakefulness and sleep, and sensory information [57]. Damage to cholinergic neurons is a critical pathological change associated with AD cognitive impairment [57]. Vascular damage is another essential component of the pathology of AD [58]. Vascular risk factors of AD, such as hypoglycemia and hypertension, lead to blood-brain barrier dysfunction during the aging process and cause damage to the neurovascular unit (NVU) [58]. Simultaneously, dysregulation in the NVU results in neurodegeneration of neuronal terminals and retrograde degeneration of cholinergic neurons, impairing the function of the blood-brain barrier and weakening the ability to clear Aβ [58]. As a result, Aβ accumulates in the brain, triggering chronic inflammation and further promoting the development of AD pathology [58]. In addition, AD pathology is associated with genetic risk factors, including Mendelian, rare, polygenic, APOE, and familial risk factors [59].

**Figure 2.**
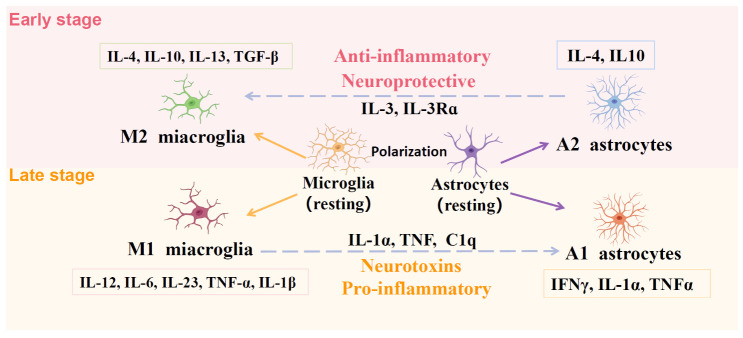
**Schematic of microglia and astrocyte-mediated polarization**. Microglia and astrocytes are usually classified into pro-inflammatory (M1/A1) or anti-inflammatory (M2/A2) phenotypes. The M1 and A1 are neurotoxic phenotypes releasing proinflammatory cytokines, including IL-12, IL-6, IL-23, TNF-α, IL-1β, IFNγ, IL-1α, and TNFα. The neuroprotective A2 and M2 phenotypes release anti-inflammatory cytokines, including IL-4, IL-10, IL-13, TGF-β, IL-4, and IL-10. Generally, during the early stage of AD, the resting microglia and astrocytes are polarized into the M2 and A2 phenotypes, secreting anti-inflammatory, and neuroprotective cytokines. However, during the late stage, the activated microglia and astrocytes are polarized into M1 and A1 phenotypes, secreting pro-inflammatory and neurotoxins cytokines.

## Polarization of microglia and astrocytes

As mentioned, microglia are broadly distributed throughout the CNS, with morphological and functional changes in response to brain injury and neurodegeneration [60]. The activated microglia can be polarized into the pro-inflammatory M1 phenotype and the anti-inflammatory M2 phenotype [61]. At the early stage of AD, microglia are activated in response to the misfolded aggregates of intracellular tau proteins and excessive Aβ accumulation [62]. Although the activated microglia at the early stage of AD is considered a neuroprotective mechanism, phagocytosis does not successfully eliminate the excessive accumulation of Aβ and abnormal tau [4]. Indeed, with the progression of AD pathologies, the microglial polarization also changes. The microglia are polarized from the neuroprotective M2 phenotypes to the neurotoxic M1 phenotypes [4]. The activated microglia at the early stage of AD are mainly M2 phenotype [4]. The M2 phenotype exerts neuroprotective effects by releasing anti-inflammatory cytokines (i.e., IL-4, IL-10, and IL-13) and elevated phagocytosis, aiding in the inflammatory response against AD pathologies [63]. At the late stage of AD, microglia polarization from M2 to M1 phenotypes occurs (Fig. 2) [5, 7]. The M1 microglia exacerbate AD pathology by releasing pro-inflammatory factors (i.e., IL-1β, IL-6, TNF-α, and IL-18) and reactive oxygen species (ROS) [4, 64].

Like microglia, astrocytes at different stages of AD exhibit different phenotypes and functions [4, 5, 65]. Under physiological conditions, the astrocytes are quiescent, maintaining brain homeostasis, regulating neurotransmitters, and preserving synaptic function and neuroimmune response [66, 67]. However, astrocytes are activated during brain injuries or neurodegenerative diseases and perform different functions [22, 68]. In the early stage of AD, the astrocytes are activated and mainly polarized into neuroprotective A2 phenotype [69]. Similarly, the A2 astrocytes have an anti-inflammatory phenotype and release anti-inflammatory cytokines in response to AD pathologies [69]. During later stages, A2 astrocytes undergo polarization to the A1 phenotype in response to the excessive accumulation of Aβ, fragmented mitochondria, oxidative stress, and neuroinflammation. The pro-inflammatory cytokines released by A1 astrocytes exacerbate AD pathology and provide positive feedback for enhanced neuroinflammation [70, 71].

## Microglia and astrocyte senescence

Cellular senescence refers to the state in which cells enter a cell cycle arrest during aging or in response to external stimuli [72]. In a senescent state, cells lose proliferative, differentiative, and functional capacities [72]. The senescence-associated secretory phenotype (SASP) represents a characteristic feature of cellular senescence [73]. Senescent cells secrete many inflammatory factors through SASP, thereby engaging in the pathological processes of neurodegenerative changes [73]. Previous studies have established the presence of senescent microglia and astrocytes in the brain tissue of AD [74, 75]. Recent research has revealed that in postmortem tissues of APP/PS1 mice and AD patients, early and sustained proliferation promotes a replicative senescence phenotype in microglia. This senescence phenotype is characterized by increased SA-β-gal activity, shortened telomeres, and aging-associated transcriptional markers [74, 76]. Inhibiting early proliferation of microglia hinders the aging process in APP/PS1 mice while suppressing the accumulation of Aβ, neuroinflammation, and synaptic injury, providing an effective therapeutic strategy to prevent subsequent pathological processes [74, 76]. In addition, astrocyte senescence exhibits characteristic features of decreased normal physiological function and increased secretion of SASP factors, contributing to Aβ deposition, tau hyperphosphorylation, and the formation of NFTs in AD [77].

Furthermore, astrocyte senescence leads to various detrimental effects, including the induction of glutamate excitotoxicity, the impairment of synaptic plasticity, the loss of neural stem cells, and the dysfunction of the blood-brain barrier (BBB) [77]. Interestingly, astrocytes show substantial accumulation around senile plaques in aged individuals. Most of these accumulated astrocytes express the age-related secretory factor interleukin-6 (IL-6), suggesting that astrocytes clustered around senile plaques possess aging characteristics [75]. Previous studies have indicated that clearance of senescent astrocytes contributes to improving cognitive dysfunction and pathological changes in tau [75]. Thus, therapeutic strategies targeting aging astrocytes may be a novel approach to treating age-related neurodegenerative diseases.

## Crosstalk between microglia and astrocytes

The cross-talk between microglia and astrocytes is implicated in the pathological progression of AD [42]. As previously described, microglia and astrocytes are essential in the immune response [78]. In both physiological and pathological conditions, astrocytes and microglia work synchronously and complementarily to maintain the normal function of the blood-brain barrier, neuroinflammation, and other cellular processes involved in brain homeostasis [78, 79]. According to previous studies, astrocyte-derived cytokines, including inflammatory cytokines and chemokines, regulate microglial function [80]. The astrocyte-derived interleukin-15 (IL-15) promotes microglial polarization to a pro-inflammatory phenotype, bridging astrocyte-microglia crosstalk. The IL-15-mediated astrocyte-microglia crosstalk elevates the production of pro-inflammatory cytokines (i.e., CD86, TNF-α, and IL-1β) and exacerbates neuronal damage following intracerebral hemorrhage [80]. In addition, astrocytes regulate microglial migration, phagocytosis, microglia-mediated synaptic pruning, and neural circuit development with interleukin-33 [81]. Intriguingly, astrocyte-derived inflammatory cytokines contribute to microglial migration and phagocytosis by increasing the permeability of the BBB and the recruitment of immune cells [78, 82].

Moreover, microglia also regulate astrocyte function and exacerbate neuroinflammation and neurotoxicity in brain injuries and neurodegenerative diseases [83, 84]. For example, previous studies demonstrated that pro-inflammatory cytokines released by activated microglia could induce astrocyte activation and determine the phenotypes of reactive astrocytes [78, 85]. Co-cultures of microglia and astrocytes significantly promoted lipopolysaccharide (LPS)-induced neurotoxic cytokine release from astrocytes, suggesting microglia enhance the neurotoxic effects of astrocytes following excessive activation [85, 86]. In normal aging and AD, the fragmented mitochondria released from the microglia contribute to the astrocyte’s polarization to the neurotoxic A1 phenotype [87]. Astrocytes compensate for the neuroimmune system dominated by microglia in acute brain injury through appropriate cross-talk between astrocytes and microglia [79]. Microglia are the first line of defense against brain injury by phagocytosing dead cells and debris and releasing anti-inflammatory cytokines [88]. Astrocytes are activated following the microglial reaction, releasing inflammatory cytokines, and forming glial scars [79, 88].

The crosstalk between microglia and astrocytes exhibits excellent potential for AD treatment [89]. A previous study shows that astrocyte-derived interleukin-3 (IL-3) could reprogram microglial function and promote microglia-mediated phagocytosis [89]. In detail, the accumulation of Aβ causes elevated expression of microglial interleukin-3 receptor alpha (IL-3Rα), a specific receptor of IL-3 [89]. The microglial IL-3Rα receptor receives the signal from the astrocyte-derived IL-3 and increases microglial motility and ability to cluster around amyloid plaques [89]. The enhanced microglial clustering and phagocytosis promote Aβ clearance and potentially alleviate AD pathologies [89]. While more studies are needed, the crosstalk between microglia and astrocytes is promising as a novel therapeutic target for AD treatment.

## Microglia and astrocyte-mediated Aβ seeding, propagation, and clearance

Although controversial, the amyloid cascade hypothesis proposes that Aβ accumulation causes various pathological changes, including neurofibrillary tangles, neuronal dysfunction, neuroinflammation, and cognitive deficits [90, 91]. Activated microglia engage in the disease-associated microglia (DAM) response [92]. Likewise, disease-associated astrocytes are activated, contributing to AD progression by polarizing towards the pro-inflammatory phenotype and exacerbating neuroinflammation [4, 5]. Recently, several studies identified new roles of astrocytes and microglia in AD [66, 90, 93].

*Aβ Seeding.* Brain-wide post-mortem studies found that Aβ deposition does not randomly form amyloid plaques throughout the brain. Instead, Aβ accumulated in a stereotyped pattern, with amyloid detected initially in the neocortex, subsequently in the allocortex, and lastly in the subcortex [91]. During this process, small amounts of aggregation components function as a protein template, and the β-sheet-rich protein structure induces the misfolding and deposition of the normally soluble protein. This process, usually referred to as seeding, contributes to Aβ propagation in AD [94, 95]. The polymerization of monomeric or oligomeric Aβ peptides causes the formation of insoluble fibrillar Aβ and amyloid plaques following Aβ propagation and seeding at new locations [91]. The polymerization process has two phases: the initial nucleation phase and the growth phase [94, 96]. During the initial nucleation phase, the formation of the putatively multimeric nucleus (“seed”) following Aβ seeding causes a rapid increase in cognate proteins that are incorporated into amyloid fibrils in AD [97]. In the growth phase, Aβ aggregation is a progressive and time-dependent process. During this process, the fragmentation of Aβ fibrils causes increased Aβ seeding, leading to a vicious cycle of progressive protein polymerization and misfolding [94, 95]. In AD, primary nucleation is a slow and time-consuming process [98]. However, the deposition of diffusible oligomeric protein is inevitable after the initial nucleation stage and progresses rapidly [91].

Previously, neurons were considered the only cell type capable of generating Aβ [18]. However, increasing evidence demonstrates that astrocytes and microglia are involved with Aβ deposition [18, 99, 100]. In AD, gliosis occurs in response to Aβ accumulation, neuronal damage, and mitochondrial dysfunction [18]. The interactions between glial cells and amyloid plaques involve a membrane protein Triggering Receptor Expressed on Myeloid Cells 2 (TREM2) [101]. Neurodegenerative diseases are closely associated with microglia, including chemotaxis, phagocytosis, and genetic variants in microglial TREM2 [101]. Previous studies reported that TREM2 deficiency caused increased Aβ seeding and impaired microglia barrier function [99, 100]. Intriguingly, TREM2-dependent microglial activity restricts amyloid plaque growth in the early AD stage but exacerbates AD progression at the late stage [99, 102]. For example, at the early stages of AD progression, TREM2 activation triggers the clustering of phagocytic CD68-positive microglia around amyloid plaques and may facilitate the clearance of Aβ [99]. However, genetic variation and deletion of TREM2 impair the microglial clustering around plaques and cause the accumulation of extracellular and intraneuronal deposits [103, 104].

In addition to microglia, astrocytes contribute to Aβ production and seeding [105]. A previous study found that pro-inflammatory factors or a small amount of Aβ could cause elevated activity of β-Site APP cleaving enzyme 1 (BACE1), APP, and β-secretase within astrocytes. This elevation of function led to increases in astrocytic Aβ production. Since astrocytes are more abundant than neurons, astrocyte-derived Aβ is an essential source of Aβ [105]. The produced Aβ42 oligomers and fibrils trigger higher levels of astrocytic BACE1 to elevate cerebral Aβ levels, thereby promoting a vicious positive feedback cycle [105]. Previous research demonstrated that the pro-inflammatory cytokines (IFNγ and TNFα), JAK2 and ERK1/2 signaling pathways, and the nuclear factor-κB (NF-κB) mediate the upregulation of BACE1 in astrocytes [106-108]. Moreover, TGF-β1 enhances Aβ production by upregulating astrocytic APP and exacerbating the burden of plaques in the brain of AD patients [109]. Notedly, activated microglia are one of the primary sources of inflammatory cytokines [110]. Thus, microglia may contribute to the positive feedback mechanism of astrocytic Aβ production. Therefore, microglia and astrocytes participate in Aβ production and seeding.

*Aβ propagation.* As mentioned, microglial activation is a neuroprotective mechanism against AD pathologies under normal conditions. However, under pathological conditions, the excessive activation of microglia accelerates AD progression by releasing pro-inflammatory cytokines and enhancing Aβ seeding [4, 5, 93, 111]. In addition to neuroinflammation and Aβ seeding, recent studies identified microglia-mediated Aβ propagation [90, 93]. Researchers transplanted Aβ from transgenic AD mice to wild-type (WT) grafts, an unaffected brain tissue. During the study, only a small number of neuronal axons from the affected brain tissue were present in the WT grafts, indicating neuronal axons are not involved in Aβ propagation [90]. Intriguingly, prominent microglia invasion from the AD tissue to the WT grafts was evident, and Aβ was identified within microglia. These findings suggest that microglia participate in Aβ propagation and contribute to seeding into unaffected brain tissue [90]. Notably, these microglia in the WT grafts were derived from the host brain tissue and had no apparent cellular death [90]. However, another study found that activated microglia establish an intimate connection with Aβ plaques, subsequently causing microglial cell death [112]. The Aβ released by dying microglia exacerbated Aβ accumulation, further confirming microglial contribution to Aβ propagation [112].

In addition, the adaptor protein apoptosis-associated speck-like protein containing a CARD (ASC) in microglia also contributes to Aβ seeding and spreading [113]. Injections of ASC specks to the hippocampus led to Aβ spreading in the AD mouse model. In contrast, ASC-deficient mice or co-application of ASC-blocking antibodies inhibited Aβ propagation and exacerbated AD progression [113]. During this process, early immune activation of IL-1β leads to the activation of NLRP3 inflammasome, triggering the assembly of ASC specks and phagocytosis by neighboring microglia to mediate further immune response [114, 115]. Thus, ASC released by microglia acts as a cross-seed for Aβ pathology, promoting Aβ aggregation and propagation in AD [113]. Aβ plaques create a unique environment that facilitates local tau seeding in dystrophic axons, leading to the propagation and formation of neuritic plaque (NP) tau at early seeding stages [116]. Furthermore, increasing microglia cluster around the amyloid plaques, elevating NP-tau seeding and spreading [111]. The astrocyte contribution to Aβ propagation remains unclear; thus, future research should aim to elucidate their role in Aβ aggregation.

*Aβ clearance.* Microglia, the core component of the cerebral immune system, play an essential role in the defense against Aβ burden. Activated microglia protect neurons by surrounding and compacting Aβ plaques to form a physical barrier, limiting the expansion of plaques and reducing neurite dystrophy [117]. Microglia interaction with Aβ plaques polarizes microglia to an anti-inflammatory phenotype and initiates microglial phagocytosis, leading to Aβ clearance [70]. Microglia promote Aβ clearance through direct interaction with PRRs [118], toll-like receptors (TLRs) [119], scavenger receptors (SR) [120], TREM2 [121], the autophagy machinery of LC3-associated endocytosis (LANDO) [122], type 1 transmembrane protein (CD33) [123] and other receptors [124]. Furthermore, the cell surface receptor of Aβ is a composite of the A scavenger receptor (SR-A) and the class B scavenger receptors, including CD36, α6β1 integrin, CD14, and CD47 [125]. A cascade of intracellular signal pathways activated by targeting these receptors can enhance the phagocytic activity of microglia to remove Aβ [126]. Besides microglial phagocytosis, microglia contribute to Aβ clearance through degradation [127-129]. For example, microglia enhance Aβ clearance by releasing amyloid-degrading enzymes, such as an insulin-degrading enzyme (IDE), epinephrine (NEP), metalloproteinases (MMPs), and lysosomal protease [128-130]. Notedly, previous research suggested that IDE and NEP contributed to the degradation of monomeric peptides but not Aβ amyloid fibrils [124, 131]. However, MMP-2 and MMP-9 are zinc-dependent metalloproteases capable of cleaving fibrillar Aβ in the brain [128]. Furthermore, the lysosome possesses endopeptidase activity and contains more than 60 hydrolases, among cathepsin B, and tripeptidyl peptidase 1, which can destabilize fibril stability and proteolyze fibrillar Aβ efficiently [130, 132]. In addition, microglial complement receptor 3 (CR3) and complement component (C3) are involved in the phagocytosis and clearance of fibrillar Aβ by microglia in vitro and in vivo [133]. Another study suggested that microglial CR3 regulates Aβ homeostasis through secreted proteolytic activity [134].

Like microglia, astrocytes respond to AD pathogenesis and are involved in Aβ clearance [54]. According to a previous study, astrocytes secreted protease MMP-2 and MMP-9 to promote Aβ clearance [135]. Increases in neprilysin and IDE enhance astrocyte-mediated Aβ clearance [136, 137]. Similarly, astrocytes are involved in Aβ clearance through the secretion of kallikrein-related peptidase 7 (KLK7), which degrades Aβ [138, 139]. In AD, KLK7 levels were reduced [138]. In addition, reactive astrocytes express multiple receptors that are involved in Aβ clearance by recognizing and combining Aβ, including the receptor for advanced glycation end products (RAGE) [140], lipoprotein receptor-related proteins (LRPs), and membrane-associated proteoglycans [141]. In addition to astrocytic Aβ production and astrocyte-mediated inflammatory response, astrocytes contribute to Aβ seeding and deposition via the glymphatic system [142]. Aquaporin-4 (AQP4), a water-selective transporter expressed in astrocytes, is essential in glymphatic system-mediated Aβ clearance [143]. In AD, the reduced expression or depolarization of AQP4 contributes to Aβ deposition [144, 145]. Our studies have found that AQP4 polarization is closely associated with astrocyte phenotype polarization. The improved astrocytic AQP4 polarization enhances Aβ clearance through the glymphatic system [146].

## Microglia and astrocyte-mediated synapse loss and pruning

Synaptic loss is a prominent feature of Alzheimer’s disease and the most robust correlate for learning and memory in AD [147]. Microglia identify and remove unnecessary neural connections, which is crucial in brain development and homeostasis [47]. Synaptic pruning mainly happens in postnatal development, wherein the brain eliminates extra synapses to refine neuronal circuits [47, 148, 149]. However, excessive microglia-mediated pruning in the adult brain induces abnormal synaptic refinement, leading to behavioral deficits associated with neurodegeneration [47, 48]. Recent studies also show that astrocytes play a role in synaptic phagocytosis [150].

### Microglia-mediated “eat me” signal pathways

The “eat me” signal is essential in microglia-mediated pruning [151]. Microglial exposure to and recognition of the “eat me” signal begins the phagocytosis of the target cells [152]. The complement C1q and C3 are the constituents of the “eat me” signal that trigger microglial engulfment of excess synapses through the activation of C3 receptors (CR3) [153, 154]. Synaptic pruning by microglia is regulated by complement-dependent mechanisms and neuronal activity. During this process, the C1q label-based synaptic pruning is essential in the selective removal of synapses by the adjacent microglia [155]. C1q is the classical complement pathway’s initial component, binding to pathogens, apoptotic cells, and synapses [156, 157]. Subsequent activation of the downstream cascade induces proteolytic cleavage of C3, promoting microglial prune complement-tagged synapses [156].

In aging and neurodegenerative diseases, excessive activation of complement-mediated synapse pruning causes synapse loss and behavioral alterations in the aging brain [158]. Previous research suggested that knocking out complement systems such as C1q, C3, or CR3 disrupted microglia-specific phagocytic pathways, consequently impairing synaptic connectivity [158, 159]. Indeed, synaptic C1q is abnormally elevated in AD, triggering early synapse loss by microglial phagocytosis [160]. Furthermore, the malfunction of the selective pruning of synapses by microglial phagocytosis strongly affected memory processes [155, 161]. Recently, studies have shown that microglia eliminate synapses in the C1q dependent-complement pathway, which strongly regulates forgetting less-active memories [161]. Overexpression of CD55 (inhibitor of complement pathways) further demonstrates that suppressing microglia phagocytosis prevented forgetting in a complement- and activity-dependent manner [161].

Beyond the complement system, exposed phosphatidylserine (PS) is another extrinsic signal representing the ‘eat-me’ signal that drives aberrant microglia-mediated synapse pruning in AD [162, 163]. During developmental pruning, localized PS exposure is a usual neuronal signal that promotes microglial engulfment through interactions with complement or other proteins [163]. The recognition of exposed PS by microglial phagocytic receptor TREM2 enables synaptic pruning associated with apoptotic cells and debris clearing [164]. In addition, perivascular secretion of phosphoprotein 1 (SPP1/osteopontin) modulates microglia-mediated synaptic engulfment in the hippocampus of AD context [162]. Furthermore, upregulation of SPP1 is required to activate C1q [162].

*Microglia-mediated ‘don’t eat me’ signal pathways*

**Figure 3.**
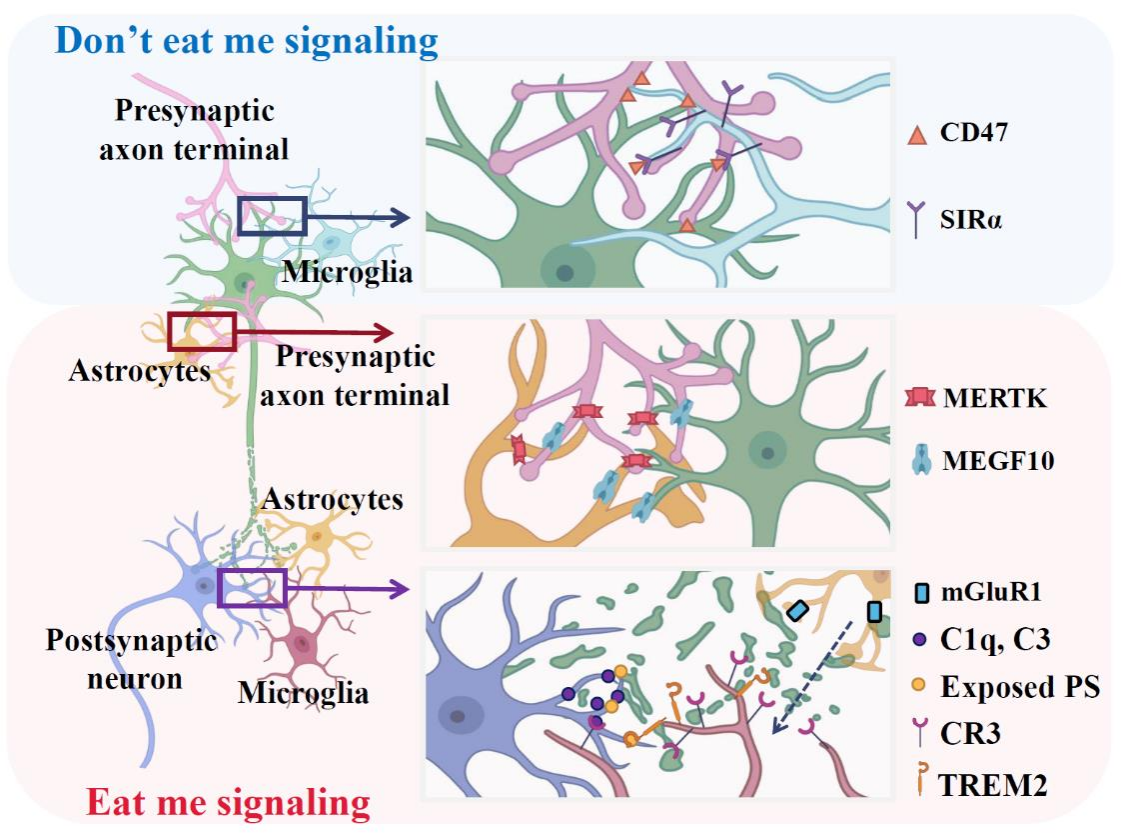
**Microglia and astrocyte-mediated “eat me” and “don’t eat me” signals**. During development, the “eat me signal” is crucial for eliminating unnecessary or inappropriate synapses. This process involves the complement system (C1q, CR3, and C3) and PS mediated by microglia, MERTK, and MEGF10 mediated by astrocytes. However, the microglial SIRα mediated “don't eat me” signal is a negative synaptic pruning regulator to avoid excessive synapse loss.

In contrast, during synaptic refinement, the ‘don’t-eat-me’ signal is the negative regulatory pathway to counterbalance the effects of ‘eat-me’ signals, preventing excessive synapse elimination (Fig. 3) [165]. CD47 is a transmembrane immunoglobulin superfamily protein that acts as a specific ‘don’t eat me’ pathway protein, interacting with its receptor signal regulatory protein-α (SIRPα), which is highly expressed on microglia during developmental peak pruning periods [166]. Previous research discovered that CD47-SIRPα interactions regulate myelin phagocytosis [47, 167]. However, recently, a similar study suggested that during postnatal development, CD47-SIRPα interaction protects synapses from aberrant pruning [168, 169]. CD47 knockout mice displayed sustainable synapse reduction with increased microglial engulfment in the dorsal lateral geniculate nucleus, suggesting the CD47-SIRPα axis limits aberrant microglial phagocytosis [170]. Furthermore, CD47 preferentially localized in more active neurons to engulf synapses, indicating the protective role of CD47 against targeting specific synaptic populations by microglia [169]. In addition, a recent study suggested the exact effect of microglial SIRPα alongside the progression of AD [168]. Using a microglia-specific SIRPα knockout mouse model, a previous study elucidated that knockout of microglial SIRPα enhances excessive synaptic loss by promoting microglia phagocytosis and exacerbates the cognitive deficits in AD [168]. In line with this, microglial SIRPα levels in the AD model have no significant changes compared with the control animal during the early AD stage. However, the microglial SIRPα significantly decreases at 5 or 8 months of age, suggesting the microglia-mediated ‘don’t eat me’ signal is essential in the cognitive impairment of AD [168].

In addition, several proteins that bind to C1q and downstream complement factors protect synapses against excessive elimination in the brain [171, 172]. Previous evidence suggested that the secreted neuronal sushi repeat protein X-linked 2 (SRPX2) acts as a complement inhibitor and binds to C1q, limiting complement activation and protecting synapses from complement-mediated elimination in the visual system and somatosensory cortex [173]. Furthermore, another study found that during the early development of the central nervous, C1q and SRPX2 interaction regulates microglia-mediated synapse pruning in the visual thalamus but not in the visual cortex [172]. Moreover, a recent study demonstrated that neuronal pentraxin 2 (Nptx2) is involved in complement activation and regulates microglia-mediated synapses in neurodegenerative diseases. Neuronal overexpression of Nptx2 sufficiently diminished complement system activation and ultimately decreased synapse loss [171]. In summary, the disruption of microglia-mediated ‘don’t eat me’ signal contributes to microglia-mediated excessive pruning of synapses and enhances cognitive impairment.

### Astrocyte-mediated microglial phagocytosis of synaptic pruning

Astrocytes also affect microglia-mediated synaptic pruning through the activated complement cascade and other proteins. The increased activation of the metabotropic glutamate receptor 1 (mGluR1) signaling facilitates synaptic C1q activation and microglial phagocytosis of hippocampal glutamatergic synapses in hippocampal CA1, inducing synaptic and cognitive impairment in AD [174]. A previous study reported that the upregulation of mGluR1 is mediated by disruption of astrocytic glutamate transporter 1 (GLT1/EAAT2) [175]. Astrocytic GLT1 expression is markedly decreased in the early stages of AD and specifically occurs in surrounding amyloid plaques [175]. Thus, astrocytic GLT1 dysfunction may contribute to the increase of C1q-mediated microglial phagocytosis of synapses in the setting of AD. Furthermore, recent findings demonstrated that blocking type 2A protein phosphatase (a key enzyme downstream of mGluR1 signaling) attenuated the microglial phagocytosis of glutamatergic synapses and cognitive deficiency in the rat model of AD [176]. In addition, selective knockout of astrocyte-apoE4 decreased microglial-dependent engulfment of synapses in the presence of tauopathy, demonstrating that astrocyte-secreted apoE4 could serve as a regulator for specific microglia receptors [177].

### Astrocytes-mediated phagocytic clearance of synapses

Although most studies focused on microglia-mediated synapse pruning, the astrocytic function is also crucial for the phagocytosis of synapses throughout the brain [150]. Astrocytes are present near Aβ plaques and are closely associated with engulfed dystrophic neurites in AD [178]. Reactive astrocytes enclose and phagocytose presynaptic dystrophy axons in the hippocampus of APP mice and AD patients, resulting in the degradation of presynaptic structures [179]. The accumulation of dystrophic presynaptic structures around Aβ plaques continues with disease progression [179, 180]. However, the reactive astrocytes only affected less than 7% of dystrophic neurites, indicating astrocyte phagocytic capacity might be weakened or impaired in AD [179, 180]. Furthermore, another study further confirmed that Aβ plaques damaged the astrocytic phagocytosis of astrocytes, thereby enhancing AD pathology [180]. According to previous studies, astrocytes actively engulf synapses *in vitro* and *in vivo* through the multiple EGF-like domains 10 (MEGF10) and c-mer proto-oncogene tyrosine kinase (MERTK) phagocytic pathways, which are closely related to neuronal activity [181, 182]. Moreover, these phagocytic receptors recognize phosphatidylserine in target debris, triggering the engulfment process [183]. Deletion of either Mertk or Megf10 in astrocytes was observed in the retention of excessive functional synapses, suggesting that Megf10 and Mertk are critical for synapse remodeling in neural circuit refinement, with potential implications for learning and memory [182]. Notedly, phagocytosis of dystrophic synapses by astrocytes is detected in AD [180]. MEGF10 binds to C1q and participates in astrocytes’ clearance of apoptotic cells [184]. Therefore, AD-related decreases in MERTK and MEGF10 expression might be related to defective astrocyte phagocytosis, inducing the aggregation of dystrophic neurites and impaired synapses [185]. Therefore, the recovery of astrocytic phagocytosis could restore neural circuits, decrease neuronal damage, and ultimately limit AD progression.

## Microglia and astrocyte-mediated demyelination, myelin debris clearance, and remyelination

The classical neuropathological hallmarks of AD are the deposition of amyloid plaques and neurofibrillary tangles in the brain, resulting in neuronal damage and synaptic loss [5]. In addition, changes to myelin structure are observed with increasing age and are more pronounced in AD [186, 187]. Generally, the reduction of white matter volume and the pathological demyelination or remyelination process contribute to decreasing axon size and internodal distance, exacerbating AD development [186]. Recent research has revealed defects in myelin lipid biosynthesis during the preclinical stages of AD, significantly contributing to the deterioration of synaptic function and cognitive decline [188]. Interestingly, myelin-formation impairment seems to be compromised even earlier than the neurofibrillary tangles that characterize AD pathology, suggesting that myelin damage may be a primary neuropathological abnormality in AD [188].

On the other hand, the deposition of Aβ in the brain of AD patients induces the deterioration of myelin phospholipids [189]. Studies have found that Aβ causes oligodendrocyte degeneration and inhibits the formation of myelin phospholipids [190]. Therefore, myelin loss in AD may involve a positive feedback loop that accelerates further neuronal loss and disease progression [189]. In the CNS, oligodendrocytes predominately synthesize myelin phospholipids [191]. Mature oligodendrocytes depend on the proliferation, migration, and differentiation of oligodendrocyte precursor cells, gradually producing multilayered lipid sheaths around the axons that lead to the myelination of neuronal axons [191]. Moreover, astrocytes and microglia release soluble factors that affect the myelination process [192].

### Microglia and astrocyte-mediated demyelination under inflammatory conditions

In AD, the excessive activation of glial cells and increased release of pro-inflammatory cytokines contribute to myelin damage [193]. The production of pro-inflammatory mediators and the prolonged activation of microglia and astrocytes lead to enhanced oligodendrocyte degeneration, thereby affecting myelination [193]. The balance between demyelination and remyelination is essential in brain damage and repair [194]. Microglia-mediated myelin debris clearance is critical for remyelination [194]. Impaired microglial phagocytosis or inadequate recruitment to the site of demyelination impedes remyelination [194]. Moreover, the proliferation and activation of microglia in AD tissues is associated with the overactivation of the colony-stimulating factor 1 receptor (CSF1R) pathway [195]. In demyelinating white matter lesions, the pro-inflammatory cytokines released by microglia stimulate the upregulation of colony-stimulating factor 1 (Csf1), resulting in neuroinflammation and neurodegeneration [196]. Previous studies have discovered that direct injection of Csf1 into the white matter of the CNS causes focal microglial proliferation and demyelination, suggesting that the activated CSF1R signaling pathway contributes to demyelination [196].

On the other hand, pharmacological depletion of microglia in a focal demyelination model delays oligodendrocyte progenitor cell (OPC) recruitment, damages myelin protein synthesis, and decreases remyelination [197]. Therefore, CSF1R inhibitor prevents microglial proliferation and activation in AD pathology, promotes new myelin phospholipid formation, and slows disease progression [195]. Early investigation has established that the increased expression of heat shock protein 60 (Hsp60) in activated microglia is detrimental to AD [198]. The HSP60 released by microglia increases the production of pro-inflammatory factors by binding to Toll-like receptor 4 (TLR-4) on the surface of OPCs, which subsequently activates the TLR4-NFκB signaling pathway and MyD88-dependent pathway to mediate OPC apoptosis and demyelination [199, 200].

In addition to microglia, astrocytes are involved in remyelination through the recruitment of microglia and the release of cytokines affecting demyelination [194]. For instance, reactive astrocyte activation contributes to inflammatory responses resulting in excessive engulfment of myelin, leading to acute damage of white matter, breakdown of the blood-brain barrier, and demyelination through the LCN2/LRP1 signaling pathway [201]. Furthermore, astrocytes are not only responsive to demyelination in the later period of inflammation but are also highly active and involved in the early progression of the lesion [202]. Reactive astrocytes exhibit hypertrophic morphology and contain damaged myelin lipids, promoting the recruitment of inflammatory cells [202]. Therefore, astrocyte hypertrophy is one of the earliest hallmarks in the demyelination process [202]. In addition, astroglia NF-κB activation contributes to maintaining brain homeostasis, which is also pivotal to neurodegeneration, causing pro-inflammatory cytokine formation and increasing excitotoxicity in AD [203]. A previous study demonstrated that NF-κB-dependent processes within astrocytes damaged oligodendrocytes and increased myelin loss [204]. Therefore, downregulation of the astrocytic NF-κB pathway and LCN2/LRP1 signaling pathway might be a promising therapy for the attenuated demyelination that occurs in AD [205].

### Microglia and astrocyte-mediated impairment of remyelination

As mentioned previously, microglia and astrocytes participate in the demyelination in neurodegenerative diseases [206]. The balance between demyelination and remyelination is crucial for brain damage and repair [194]. Interestingly, microglia are also critical in the clearance of brain toxins, which is essential for remyelination [207]. In response to Aβ accumulation, the microglia are activated to eliminate Aβ burdens through microglial phagocytosis [208]. However, the ability of microglia to remove toxic substances from the brain is reduced during the late stage of AD, inducing myelin damage in AD events [209]. The phagocytic clearance of myelin debris resulting from lesion damage is critical for the remyelination of axons and the reconstitution of neuronal circuits [194, 210]. Insufficient or delayed debris clearance hinders remyelination and impedes regeneration [194, 210].

CX3CR1 signaling has been identified as a critical checkpoint regulator for microglial activation in AD [211]. Loss of CX3CR1 impairs microglial function, enhancing the oxidative stress response and dysregulated pro-inflammatory activation [212]. *Cx3cr1* deficiency impairs the uptake of and degradation of fibrillar Aβ by microglia, resulting in increased accumulation of neurotoxic Aβ species [212]. Furthermore, in *Cx3cr1*-deficient mice, microglial clearance of myelin debris is impaired, affecting the integrity of axons and myelin sheaths and preventing myelin regeneration, indicating a critical role of CX3CR1 in myelin debris removal [213]. These findings suggest the significance of CX3CR1 signaling in microglia-mediated myelin debris through phagocytosis in remyelination and AD pathology.

NOX4, a major isoform of the NADPH oxidase, promotes the generation of reactive oxygen species and exacerbates cognitive impairment and memory loss in AD [214]. Recent studies found that the loss of Nox4 contributed to myelin regeneration by enhancing the microglia-mediated myelin debris clearance and increasing oligodendrocytes’ production of nutrient factors [215]. Consistent with previous research, inhibiting or knocking out Nox4 decreased amyloid load and oxidative stress in APP/PS1 mice, alleviating synaptic and memory dysfunction [216]. Thus, limiting the level of Nox4 may be a potential target for promoting myelin regeneration in AD pathology.

Additionally, astrocytes are also involved in impaired remyelination. Endothelin-1 (ET-1) is highly expressed in reactive astrocytes after demyelination, drastically decreasing the rate of remyelination [217]. Mechanistically, ET-1 acts as an endogenous inhibitor of remyelination by reducing the Jagged1 expression in reactive astrocytes and enhancing Notch activation in OPCs during remyelination. Inhibition of ET-1 signaling in the demyelinated lesion inhibits Notch activation and enhances remyelination [217]. These findings suggest that astrocytes are crucial for remyelination in AD in addition to microglia.

**Figure 4.**
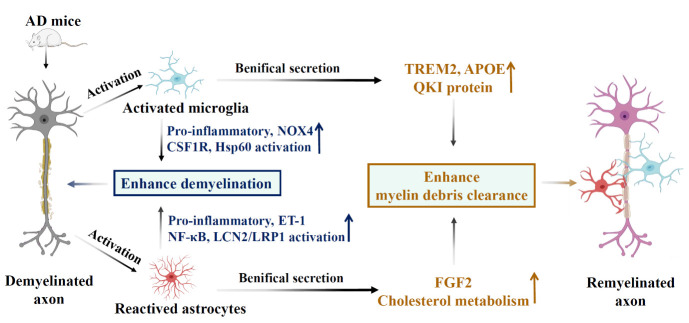
**Microglia and astrocytes are involved in phagocytosis and remyelination**. AD pathology leads to demyelination and fragmentation of myelin, consequently leading to the activation of microglia and astrocytes. These activated cells release pro-inflammatory factors (NOX4, ET-1) and upregulate the activity of CSF1R, Hsp60, NF-κB, and LCN2/LRP1, exacerbating myelin loss. However, activated microglia and astrocytes also promote TREM2, APOE, and QKI expressions, which support the clearance of myelin fragments and enhance remyelination.

### Microglia and astrocytes promote myelin debris clearance and remyelination

Currently, a wealth of research has provided evidence that activating TREM2 on microglia promotes the clearance of myelin debris and remyelination in neurodegenerative diseases (Fig. 4) [218-220]. TREM2 is necessary for the coordinated transcriptional process in microglia in response to prolonged demyelination, the clearing of damaged myelin, and the secretion of nutritional factors that support oligodendrocyte differentiation or survival [220]. Furthermore, previous studies found that TREM2-deficient microglia fail to remove damaged myelin, resulting in the accumulation of myelin debris and inhibition of the differentiation and remyelination of oligodendrocyte precursor cells [220, 221]. Recent research found that AL002a treatment can activate TREM2-dependent intracellular pathways in vitro and in vivo, potentiating microglia-mediated myelin phagocytosis and degradation [218]. Another study using a focal demyelination model in the brain demonstrated that TREM2 is crucial for efficient myelin debris uptake and remyelination following demyelinating injury [222]. TREM2 on microglia is a phospholipid-sensing receptor during demyelination. It maintains microglial phagocytic activity and binds to myelin debris with subsequent engulfment and clearance [220].

Similarly, TREM2 is required for lipid droplet biogenesis, allowing remyelination [223]. In AD pathology, TREM2-deficient microglia fail to remove myelin cholesterol, inducing pathogenic lipid accumulation [219]. Like TREM2, cholesteryl ester accumulation also occurs in APOE-deficient glial cells, resulting in damaged brain cholesterol transport [219]. Intriguingly, APOE upregulation depends on TREM2 expression, suggesting apoE-TREM2 interaction in microglia is essential for modulating phagocytosis and cholesterol transport of APOE [224]. Thus, the enhancement of TREM2 function may selectively alleviate cholesteryl ester accumulation in microglia, possibly offering therapeutic benefits in AD [219].

Recent studies on demyelinating diseases have found that the phagocytic activity of microglia is regulated by the expression of QKI protein (KH domain containing RNA binding) [225, 226]. Loss of the Qki gene impairs the microglia-mediated clearance of myelin debris and affects microglia phagocytic activity, inhibiting oligodendrocyte maturation and delaying remyelination [226]. Early studies have identified Qki as a gene exclusively expressed in glial cells, critical for promoting oligodendrocyte differentiation, moderating the expression of myelin basic protein, and participating in myelin phospholipid formation [227, 228]. However, in AD pathology, Qki expression increases with the severity of the disease [227]. Notably, current studies only explore the expression of Qki in oligodendrocytes and astrocytes of sporadic AD patients [227, 229]. Therefore, further investigation is needed to determine how the upregulation of Qki mediates the microglial phagocytic activity and remyelination in AD pathology.

Astrocytes and microglia differ in spatiotemporal phagocytic activity [230]. Astrocytes eliminated debris and remodeled synapses later than microglia but persisted longer, suggesting that astrocytes have a distinct role in different phagocytic mechanisms from microglia [230]. Under physiological conditions, either microglial ablation or microglial dysfunction can induce activation of astrocyte phagocytosis, suggesting astrocytes have the potential to support and compensate for the phagocytic clearance system predominated by microglia [231]. However, the AD model has not investigated microglial compensatory phagocytosis mediated by astrocytes.

During adulthood, astrocytes are the primary source of cholesterol in the CNS, and significant disturbances in brain lipid metabolism occur in AD, particularly in lipids abundant in myelin [232, 233]. It has been previously demonstrated that myelin instability contributes to AD progression [232]. Astrocytes regulate remyelination through cholesterol metabolism [233, 234]. Dietary cholesterol promotes remyelination, partially by affecting the astrocyte phenotype or the secretion of fibroblast growth factor 2, which supports remyelination [235]. Furthermore, astrocytes directly impact the clearance of myelin debris by expressing angiopoietin-like 4 (ANGPTL4), an inhibitor of lipoprotein lipase, facilitating myelin debris clearing [236]. Strongly decreased expression of ANGPTL4 in astrocytes could prompt microglia to sufficiently clear myelin debris, thereby achieving remyelination [236]. Recent studies have demonstrated that conditional repression of muscarinic signaling in OPCs augments myelin formation, restores myelin renewal, and improves cognitive deficits in AD mice [237]. Therefore, enhancing myelin formation may hold therapeutic potential for alleviating AD-related cognitive impairment [237]. However, the correlation between the observed lipid imbalance and demyelination remains unclear in AD pathology. Thus, additional studies should be conducted to elucidate lipid-dependent myelin homeostasis and discover new therapeutic targets for AD-related myelin instability.

Furthermore, the interplay between OPCs, oligodendrocytes, and astrocytes mediates remyelination. During the pathological process of AD, oligodendrocytes, and OPCs are highly susceptible to factors such as Aβ, oxidative stress, and neuroinflammation [238]. Moreover, in the early stages of AD pathology, functional impairments and demyelination of oligodendrocytes and OPCs have been detected, and these changes progressively worsen as the pathological process advances, eventually leading to the progressive decline and loss of neurons [238]. Following brain injury, OPCs migrate along cerebral blood vessels to the relevant brain regions and differentiate into myelin-forming cells or participate in remyelination after injury [239]. Interestingly, when perivascular astrocytes surround OPCs, their migration along the blood vessels is impeded [240]. Specifically, axon guidance factors Sema 3a and Sema 6a secreted by perivascular astrocytes relieve the inhibitory effect of endothelial cells on OPC differentiation and induce detachment of OPCs from the vascular surface [240]. These findings suggest that astrocyte-OPC interactions are essential in oligodendrocyte-mediated remyelination.

## Pharmacological approaches targeting glial cells in AD

*Galantamine.* Galantamine treatment has been used to inhibit excessive astrocyte activation, alleviate the release of pro-inflammatory cytokines (e.g., TNF-α and IL-6), and alleviate hippocampal Aβ accumulation [241]. Early treatment with galantamine in the pre-plaque stage enhances microglia function, promotes Aβ clearance, reduces insoluble Aβ in the brain, and inhibits the production of pro-inflammatory cytokines [242]. Indeed, synthetic galantamine has received regulatory approval in more than 20 counties in recent decades [243]. As a specific, competitive, and reversible inhibitor of acetylcholinesterase and an allosteric modulator at nicotinic cholinergic receptor sites, galantamine potentiates cholinergic nicotinic neurotransmission and promotes cholinergic function [244]. Therefore, galantamine has significant clinical applications in preventing AD [241, 242].

*Senolytics.* Senolytics, endowed with the remarkable capability to eradicate senescent cells selectively, have garnered substantial support in AD neuropathology, as evidenced by empirical findings from preclinical trials [245, 246]. Significantly, the combination of dasatinib (D) and quercetin (Q) (D+Q) is considered the prototypical senolytic treatment, which targets multiple types of senescent cells and has been demonstrated to effectively clear senescent cells and ameliorate various chronic diseases in preclinical models [245]. For example, senolytic treatment selectively eliminates OPC senescence in the plaque environment in AD mice, thereby improving Aβ plaque-associated inflammation and cognitive impairments [247]. Additionally, robust and widespread induction of senescent cells occurs in the brain following injury, particularly in microglia and astrocytes [248]. Senolytic therapy effectively eliminates senescent cells, reducing the expression of SASP factors, decreasing neurodegeneration, and correcting deficits in reference memory, recognition memory, and depressive behaviors [248]. Notably, preliminary results from initial clinical trials of senolytic treatment for AD support its safety, tolerability, and feasibility and suggest that astrocytes and Aβ may be particularly effective targets for therapy [249].

*Palmitoylethanolamide.* N-palmitoylethanolamide (PEA) is a non-endogenous endocannabinoid-like lipid mediator produced by astrocytes belonging to the N-acylethanolamine phospholipids class [250]. PEA, especially in ultra-micronized formulation (um-PEA), exhibits a protective role in treating neuroinflammation and neurodegenerative diseases and is a potential therapeutic molecule for AD [251]. PEA reduces the aggregation of Aβ and the phosphorylation of tau protein, promoting the normalization of astrocytic function. This normalization of astrocytic function decreases the release of the glutamate-aspartate complex and alleviates neuroinflammation, thereby improving hippocampal CA1 neuron survival [252]. Similarly, a previous study has found that continuous treatment of AD mice with 100 mg/kg/day of um-PEA for three months significantly reduces the glutamate-aspartate complex, inhibits neuroinflammation and oxidative stress response, and improves cognitive deficits [250]. Moreover, studies have demonstrated that PEA *in vitro* and *in vivo* has a significant beneficial effect on Aβ-induced neurodegeneration, not only by directly affecting neurons but also by counteracting harmful effects of astrocyte dysfunction, further improving the pathological symptoms of AD [253].

*N-methyltransferase inhibitors.* Neuronal histamine is involved in developing cognitive impairment in AD [254]. However, increasing histamine levels is a promising strategy to enhance cognitive function in AD patients. Inhibiting astrocyte histamine N-methyltransferase (HNMT) elevates histamine levels [255]. HNMT inhibitors increase histamine levels, affecting cognitive function, neural plasticity, neuron survival, and Aβ degradation [256]. Oral metoprine has been demonstrated to increase histamine levels, improving cognitive function in AD patients by inhibiting HNMT and increasing histamine levels in the brain [257]. Recently, virtual screening of FDA-approved drugs based on drug repurposing found that drugs with a high affinity for HNMT benefit AD treatment [258]. For example, a previous study screening a database of 185 FDA-approved medications for treating neurological diseases found dihydroergotamine and vilazodone targeting HNMT are promising in AD treatment [258]. Thus, inhibiting histamine N-methyltransferase in astrocytes offers the possibility of enhancing histamine’s beneficial effects in AD patients’ brains, such as improving cognitive function, promoting neural plasticity, and facilitating Aβ peptide degradation.

*Resveratrol.* Resveratrol is a natural polyphenolic compound closely related to regulating neuroprotective molecules and glial function [259]. Resveratrol is an effective activator of SIRT1, linking to the modulation of energy balance (NAD/NADH) and gene transcription, and may prevent AD similar to caloric restriction [260]. Resveratrol protects neurons from toxins and damage associated with neurodegenerative diseases by activating SIRT1. In addition, resveratrol possesses anti-inflammatory properties, preventing Aβ-induced neuronal inflammation and toxicity by inhibiting NF-κB signaling and p38 MAPK signaling in microglia and astrocytes [259, 260]. Clinical trials have shown that resveratrol maintains the integrity of the BBB by reducing MMP9 and activating microglia to produce adaptive immune responses, which may have clinical benefits for AD subjects [261]. Additionally, the antioxidant effects of resveratrol reduce the production of Aβ by inhibiting the activation of ROS [260]. Long-term treatment with resveratrol has been demonstrated to prevent changes in metabolism, oxidation, inflammation, senescence parameters, and neuroglial markers associated with aging [262]. Resveratrol stimulates aging astrocytes to resist glial cell toxication stimuli by upregulating core signaling pathways and cellular homeostasis, including adenosine receptors, nuclear factor erythroid-derived 2-like 2 (Nrf2), heme oxygenase 1 (HO-1), SIRT1, and phosphoinositide 3-kinase (PI3K), thereby attenuating the inflammatory process [263]. Furthermore, evidence indicates that resveratrol inhibits the pro-inflammatory signaling pathway dependent on the activation of adenosine receptors, participating in the anti-inflammatory activity of astrocytes [259]. Thus, given the close association between the pathology of AD and aging, the protective mechanism of resveratrol in astrocytes against inflammation through adenosine receptors in AD merits further exploration.

Presently, studies have evaluated resveratrol’s efficacy in treating AD [264]. For instance, a double-blind, placebo-controlled, randomized study assessed the impact of resveratrol on 64 patients with mild AD [265]. However, research findings indicated that resveratrol and its primary metabolites can cross the blood-brain barrier, resulting in adverse effects such as weight loss, nausea, and diarrhea [265]. Additionally, the resveratrol-treated group exhibited more pronounced brain volume loss [265]. Nevertheless, the evidence regarding resveratrol in clinical trials remains relatively weak [264, 265]. Most human studies have found an association between the consumption of resveratrol-rich foods and a decreased incidence or prevalence of AD, along with improvements in learning, memory, visual abilities, spatial orientation, and social behavior [266]. Therefore, further clinical trials are recommended to validate the neuroprotective effects of resveratrol and explore its potential mechanisms of action.

*Other pharmacological approaches*. Moreover, additional pharmacological interventions targeting the pathophysiology of AD have shown promising advancements on the clinical front. Minocycline, an anti-inflammatory tetracycline-class drug, reduces the accumulation of Aβ and attenuates microglia activation by inhibiting the NLRP3 inflammasome [267]. However, it is noteworthy that the therapeutic targeting of inflammation by minocycline did not delay the progression of cognitive impairment in AD patients, thus necessitating further research [268]. Additionally, idebenone, a free radical scavenger, similarly attenuates the pro-inflammatory response of microglia to Aβ [269, 270]. With favorable permeability across the blood-brain barrier and the capacity to exert multiple actions on diverse molecular targets, idebenone stands out as a highly promising therapeutic drug, holding immense potential for clinical applications [271, 272].

## Non-pharmacological treatment

*Exercise.* Evidence demonstrates that physical exercise provides a multifaceted benefit for alleviating AD by reducing Tau hyperphosphorylation, Aβ accumulation, and glial cell-mediated neuroinflammation [273, 274]. Exercise has been found to significantly increase the number of brain-derived neurotrophic factor (BDNF)-positive cells in the cerebral cortex and hippocampus, suppress excessive microglia activation, and effectively prevent neuron damage in aging mice [275, 276]. Furthermore, physical exercise upregulates the expression of TREM2 and SR-A, enhancing the positive modulating effect of microglia [277]. In recent years, emerging studies found that exercise promoted the transformation of activated microglia from the M1 phenotype to the M2 phenotype, reducing Aβ deposits and oxidative stress and improving hippocampal-dependent cognitive function at the early AD stage [278, 279]. Furthermore, exercise promotes the AQP4 polarization in astrocytes, enhancing the astrocyte AQP4-mediated glymphatic system’s efficiency in removing Aβ and abnormal tau clearance [280].

However, despite the encouraging results mentioned above, it is necessary to emphasize the presence of conflicting research findings. Short-term treadmill exercise has been found to increase the number of activated microglia in the hippocampal region of aged Tg18 female mice and elevate pro-inflammatory cytokines levels such as IL-1β and IL-1 [281]. Additionally, increased levels of C-terminal insoluble tau protein and tau phosphorylation have been observed following exercise [281]. These findings suggest that while exercise may positively impact AD patients, alterations in brain function may require adjustments based on the duration of exercise as well as the frequency, intensity, and type of exercise during the intervention period.

*Photobiomodulation.* Photobiomodulation (PBM) is a potential therapeutic strategy for AD, which utilizes low-power laser and near-infrared spectra to activate beneficial biological responses in cells [282, 283]. PBM therapy operated in pulsed or continuous wave modes [284]. Previous studies have revealed the beneficial effects of both pulsed light and continuous wave PBM therapies on the brain [284]. The optimal therapeutic parameters of brain PBM therapy depend on various factors, including wavelength, fluence, power density, repetition rate, treatment time, and mode of light transfer (i.e., continuous wave or pulsed light) [284]. Considerable investigations have shown the biological responses of glial cells induced by PBM therapy and its impact on Aβ clearance [282, 285]. Our previous study found PBM therapy with 808 nm low-level laser significantly enhanced the expression of IL-3 in astrocytes and IL-3Rα in microglia, resulting in recruiting microglia surrounding amyloid plaques and improving Aβ clearance [5]. Moreover, treatment with 808 nm low-level laser modulates the phenotype of microglia and astrocytes, converting them from a neurotoxic M1/A1 phenotype to a neuroprotective M2/A2 phenotype and inhibiting neuroinflammatory reactions [5]. In another study, PBM decreases vascular-associated microglia and promotes angiogenesis, attracting microglia to Aβ deposits and further reducing brain Aβ levels, ultimately leading to improved memory and cognitive function [282]. Similarly, PBM with pulsed light can also activate and induce morphological alterations of microglia in the primary visual cortex, significantly reducing Aβ burden and tau phosphorylation [286]. Notably, as mentioned previously, the beneficial effects of PBM therapy depend on multiple parameters. Thus, more studies are needed to define the optimal parameter for AD treatment and glial cell regulation.

*Low-intensity pulsed ultrasound therapy.* Low-intensity pulsed ultrasound (LIPUS) is an effective and non-invasive method for treating dementia [287]. LIPUS treatment inhibits chronic inflammatory responses in microglia of AD mice and enhances endothelial nitric oxide synthase, thereby reducing Aβ accumulation and neuroinflammation [288]. However, although LIPUS treatment reduced the total number of activated microglia, microglia phagocytosis for Aβ was significantly enhanced, indicating that the mechanism of LIPUS-induced reduction in Aβ plaque load may involve multiple factors that require further exploration [288]. In addition, previous research has shown that LIPUS treatment boosts the production of BDNF in astrocytes, regulating the development and function of neural circuits [289, 290]. Therefore, further research is needed to elucidate the role of astrocytes in LIPUS-mediated cognitive improvement in AD pathology.

**Figure 5.**
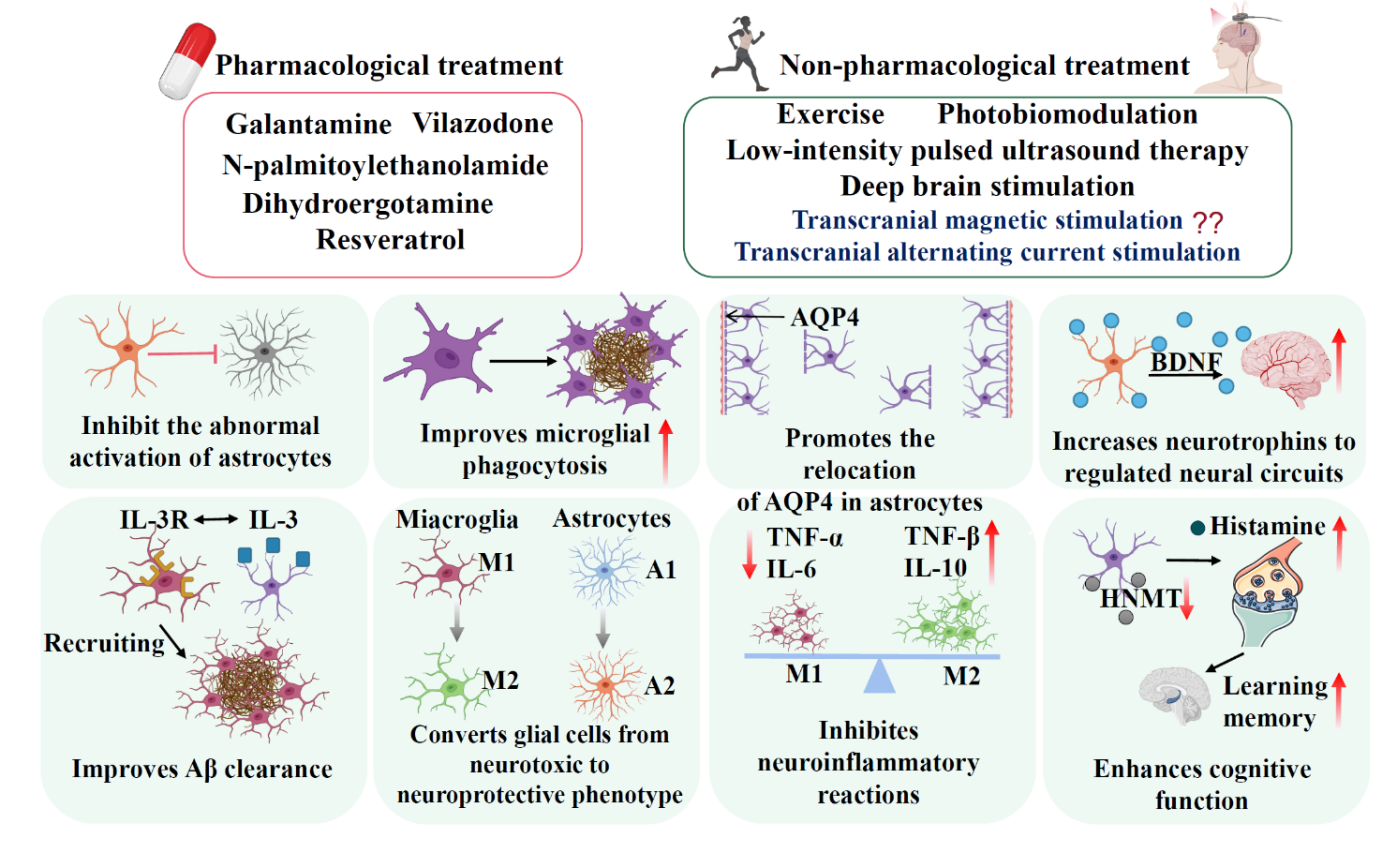
**Pharmacological and non-pharmacological treatments targeting microglia and astrocytes in AD**. Pharmacological therapies, including galantamine, Vukazidibem, N-palmitoylethanolamide, Dihydroergotamine, Resveratrol, and non-pharmacological therapies, including exercise photobiomodulation, low-intensity pulsed ultrasound therapy, and deep brain stimulation, targeting glial cells have exhibited therapeutic potential in AD. Although recent studies have shown that transcranial magnetic stimulation (TMS) and transcranial alternating current stimulation (tACS), the other widely applied non-invasive treatment for brain diseases, have the potential to slow down AD progression, the impact of TMS and tACS on glial cells require further investigation.

*Deep brain stimulation.* Deep brain stimulation (DBS) has emerged as a potential method for improving cognitive function in AD through the modulation of neural circuits [291]. Compared to non-invasive neural modulation strategies, invasive DBS through implanted electrodes targets specific brain areas and provides real-time EEG-neurofeedback, resulting in better treatment effects [292]. Chronic fornix DBS treatment significantly reduced amyloid deposition in the hippocampus and cortex, decreased activation of astrocytes and microglia, and promoted neurogenesis [293]. Interestingly, the rats treated with DBS showed a decrease in the number of abnormally activated astrocytes and morphological changes, indicating that DBS contributes to restoring the balance of the nervous system [293]. Clinical trials also found that DBS targeting the fornix helps to regulate cognitive ability and stabilize or attenuate memory impairment in mild AD patients [292, 293]. Unfortunately, current DBS therapy targeting the fornix for AD only improves episodic memory in a subset of patients [294]. Because the impact of DBS therapy on memory impairment may vary depending on the specific brain targets stimulated, DBS parameter settings, pre-existing brain states, and the specific memory processing stage are evaluated (such as encoding or retrieval) [295, 296]. Additionally, there are differences among patients participating in studies and between studies regarding age, severity of symptoms, possible current brain states, genetic backgrounds, and AD etiology [295]. Consequently, obtaining consistent outcomes of DBS therapy efficacy between patients and studies is challenging. Overall, fornix DBS appears to be a promising intervention for memory impairment in AD patients, but further research is still needed.

## Conclusion

This review revealed the dual role of astrocytes and microglia in AD pathology. Specifically, in the acute response stage of AD, microglia and astrocyte-mediated neuroinflammation contribute to restoring intra-organism balance. Neuroglial activation can prevent AD progression by releasing anti-inflammatory cytokines to mediate Aβ clearance and prevent excessive synaptic engulfment and demyelination. However, in the late stage, excessive or abnormal microglia and astrocyte activation may further induce Aβ aggregation, synaptic loss, and impair remyelination. In fact, the dual function of neuroglia in the progression of AD pathogenesis is controlled by specific inflammatory cytokines that determine their functional phenotype (Table 1).

**Table 1:** Microglia and astrocyte-mediated Aβ clearance and accumulation.

Outcome	Cell	Mediators	Functions/Activities	References
**Aβ aggregation**	Microglia	TREM2	TREM2 (usually R47H) variation interferes with microglial function and promotes dystrophic neurites around plaques, affecting the microglial phagocytic activity.	[103, 104]
**Aβ aggregation**	Astrocytes	BACE1, TGF-β1	BACE1 or TGF-β1 induces the upregulation of APP in astrocytes, enhancing the burden of plaques.	[105, 109]
**Aβ aggregation**	Astrocytes	AQP4	Perivascular AQP4 polarization induces misfolding of Aβ and impairs Aβ clearance.	[143, 144]
**Aβ aggregation and propagation**	Microglia	ASC specks	IL-1β activates NLRP3 inflammasome and triggers microglia-mediated ASC specks to assemble, resulting in the increased spreading and deposition of Aβ.	[45, 114, 115]
**Aβ clearance**	Microglia	Anti-inflammatory cytokines	Microglia-activated M2 phenotypes induce increased anti-inflammatory cytokines, restraining pro-inflammatory cytokines and leading to the clearance of Aβ.	[70]
**Aβ clearance**	Microglia	Receptors	Several receptors contribute to the microglia-enhanced phagocytic activity, including PRRs, TLRs, SR, TREM2, LANDO, CD33, and other receptors.	[118-124]
**Degrade Aβ fibrils and oligomers**	Microglia	Amyloid-degrading enzymes	Microglia releases amyloid-degrading enzymes, including IDE, NEP, MMPs, and lysosomal protease, destabilizing fibril stability and proteolyzing fibrillar Aβ efficiently to promote the clearance of Aβ.	[128-130]
**Degrade Aβ fibrils**	Astrocytes	Amyloid-degrading enzymes	Astrocytes secreted protease MMP-2, MMP-9, neprilysin, IDE, and KLK7 capable of degraded fibrillar Aβ pathology that alleviates the toxic effects of peptide deposition.	[135-139]
**Aβ clearance**	Astrocytes	Receptors	Reactive astrocytes express multiple receptors, including RAGE, LRPs, and membrane-associated proteoglycans, combining and clearing Aβ.	[140, 141, 299]

We also discussed potential interventions targeting microglia and astrocytes in AD pathology, including drug therapies, exercise, photobiomodulation, low-intensity pulsed ultrasound therapy, and deep brain stimulation (Fig. 5). Although recent studies have shown that transcranial magnetic stimulation (TMS) and transcranial alternating current stimulation (tACS), the other widely applied non-invasive treatments for brain diseases, have the potential to slow down AD progression, the impact of TMS and tACS on glial cells requires further investigation [297, 298]. Microglia and astrocytes are essential in multiple processes involved in the initiation and development of AD. Therefore, targeting microglia and astrocytes may be a promising approach to alleviate neuronal damage and AD pathologies.
